# Attention to visual motion suppresses neuronal and behavioral sensitivity in nearby feature space

**DOI:** 10.1186/s12915-022-01428-7

**Published:** 2022-10-05

**Authors:** Sang-Ah Yoo, Julio C. Martinez-Trujillo, Stefan Treue, John K. Tsotsos, Mazyar Fallah

**Affiliations:** 1 Department of Psychology, York University, Toronto, ON M3J 1P3 Canada; 2 Department of Electrical Engineering and Computer Science, York University, Toronto, ON M3J 1P3 Canada; 3 Centre for Vision Research, York University, Toronto, ON M3J 1P3 Canada; 4 Department of Physiology and Pharmacology, and Psychiatry, Western University, London, ON N6A 5B7 Canada; 5 Cognitive Neurophysiology Laboratory, Robarts Research Institute, Schulich School of Medicine and Dentistry, Western University, London, ON N6A 5B7 Canada; 6 Cognitive Neuroscience Laboratory, German Primate Centre - Leibniz Institute for Primate Research, 37077 Goettingen, Germany; 7 Faculty for Biology and Psychology, University of Goettingen, 37073 Goettingen, Germany; 8 Leibniz ScienceCampus Primate Cognition, 37077 Goettingen, Germany; 9 Bernstein Center for Computational Neuroscience, 37077 Goettingen, Germany; 10 Vision: Science to Application, York University, Toronto, ON M3J 1P3 Canada; 11 Center for Innovation and Computing at Lassonde, York University, Toronto, ON M3J 1P3 Canada; 12 School of Kinesiology and Health Science, York University, Toronto, ON M3J 1P3 Canada; 13 Department of Human Health and Nutritional Sciences, University of Guelph, Guelph, ON N1G 2W1 Canada

**Keywords:** Feature-based attention, Attentional surround suppression, Motion processing, Selective Tuning model

## Abstract

**Background:**

Feature-based attention prioritizes the processing of the attended feature while strongly suppressing the processing of nearby ones. This creates a non-linearity or “attentional suppressive surround” predicted by the Selective Tuning model of visual attention. However, previously reported effects of feature-based attention on neuronal responses are linear, e.g., feature-similarity gain. Here, we investigated this apparent contradiction by neurophysiological and psychophysical approaches.

**Results:**

Responses of motion direction-selective neurons in area MT/MST of monkeys were recorded during a motion task. When attention was allocated to a stimulus moving in the neurons’ preferred direction, response tuning curves showed its minimum for directions 60–90° away from the preferred direction, an attentional suppressive surround. This effect was modeled via the interaction of two Gaussian fields representing excitatory narrowly tuned and inhibitory widely tuned inputs into a neuron, with feature-based attention predominantly increasing the gain of inhibitory inputs. We further showed using a motion repulsion paradigm in humans that feature-based attention produces a similar non-linearity on motion discrimination performance.

**Conclusions:**

Our results link the gain modulation of neuronal inputs and tuning curves examined through the feature-similarity gain lens to the attentional impact on neural population responses predicted by the Selective Tuning model, providing a unified framework for the documented effects of feature-based attention on neuronal responses and behavior.

**Supplementary Information:**

The online version contains supplementary material available at 10.1186/s12915-022-01428-7.

## Background

Attention, defined as the selection and modulation of information processing in the brain, allows sensory systems to deal with information processing overload [1]. Attention can be allocated to a region of space (spatial attention) or to object features (feature-based attention). Feature-based attention facilitates the processing of the attended feature relative to unattended ones [2–11]. Electrophysiological studies in behaving monkeys have shown that feature-based attention can modulate the responses of sensory neurons to visual stimuli [4, 12]. This effect is described as a monotonic change in the gain of neuronal responses following the feature-similarity gain principle [4]. In these studies, feature-based attention enhances the response gain of the neurons selective for the attended feature, an effect that grows smaller to become a suppression as the neuron’s preferred feature differs from the attended feature [4, 8]. On the other hand, a computational model, Selective Tuning model of visual attention [1, 13, 14], has predicted a non-linear effect of feature-based attention (feature-based surround suppression) which has been corroborated by human studies [15–22]. When a feature is attended, responses to nearby unattended features in feature space are suppressed. Since the attended stimuli often have other stimuli nearby in real scenes (the *context problem* [14]), the Selective Tuning model ameliorates contextual interference via top-down attention, e.g., suppressing responses to stimuli in the neighborhood of the attended stimulus. The context problem and the Selective Tuning model’s solution can apply to space, features, or objects [1, 13, 14]. The link between the gain effects observed in single neurons and the non-linearities predicted by computational modeling and observed during behavior remains unclear.

Behavioral studies using a visual motion attentional cueing paradigm found that participants’ performance decreased as the direction offset between a cue and a target stimulus became greater; however, performance gradually recovered when the offset was larger than 90° [20, 21], indicating feature-based surround suppression in motion processing. On the other hand, a previous neurophysiological study of feature-based attention has shown that tuning curves of direction-selective neurons in the middle temporal area (MT) show mainly gain changes [4]. This study, however, used moving random dot patterns (RDPs) positioned in different hemifields and recorded from area MT.

neurons with receptive fields (RFs) localized to the contralateral visual hemifield. It is possible that the lack of interference (context within the Selective Tuning model’s framework) due to the stimuli being far away and the relatively small attentional modulation of responses documented in these conditions is insufficient to produce the feature-based surround suppression predicted by the Selective Tuning model and observed in behavioral studies. The present study aims at clarifying these issues.

First, we measured the activity of direction-selective neurons in MT and medial superior temporal (MST) visual cortical areas of macaque monkeys. We obtained tuning curves of direction-selective neurons by placing two moving RDPs within a neuron’s RF—one RDP always moved in the neuron’s preferred direction (preferred pattern) and the other moved in one of twelve different directions (tuning pattern). In different trials, we instructed the animals to direct attention either to the fixation point (fixation condition) or to one of the RDPs (attend-preferred and attend-tuning conditions). We found that during fixation, neuronal and population tuning curves were well fitted by a single Gaussian curve with positive gain. However, when the animals attended to the preferred pattern, neuronal tuning curves exhibited a suppressive surround. Here, response profiles were better described by adding a second wider Gaussian function with negative gain. We modeled the feature-based surround suppression by the additive interaction of the two Gaussian fields representing excitatory and inhibitory input fields into a neuron. Feature-based attention disproportionally increases the gain of the inhibitory wider relative to the excitatory narrower input field producing a suppressive surround.

In the behavioral experiment, we measured behavioral correlates of this feature-based attentional suppressive effect on motion repulsion, a perceptual illusion arising from an overestimation of the directional difference between the two superimposed motion surfaces [23–25]. Studies demonstrated that feature-based attention affects the magnitude of motion repulsion [26, 27]. Specifically, directing attention to one motion direction reduces motion repulsion, suppressing the inhibitory effect from the other (unattended) motion direction. We hypothesized feature-based surround suppression would reduce motion repulsion to a greater degree if the attended and the unattended motion directions are similar. Furthermore, using motion repulsion could provide a more sensitive behavioral measure than the electrophysiological study where animals’ task performance is high so we would not expect to see apparent feature-based surround suppression at the behavioral level. We measured motion repulsion under different attentional conditions (focused vs. divided attention) while the directional difference between two superimposed motion surfaces varied. Consistent with surround suppression, motion repulsion was minimized when one motion direction was attended while the unattended direction was close to the attended one compared with greater direction differences. These results support the Selective Tuning model’s prediction that surround suppression in feature-based attention reduces interference from the unattended feature in nearby feature space, consequently decreasing motion repulsion.

## Results

### Neurophysiology

**Fig. 1:**
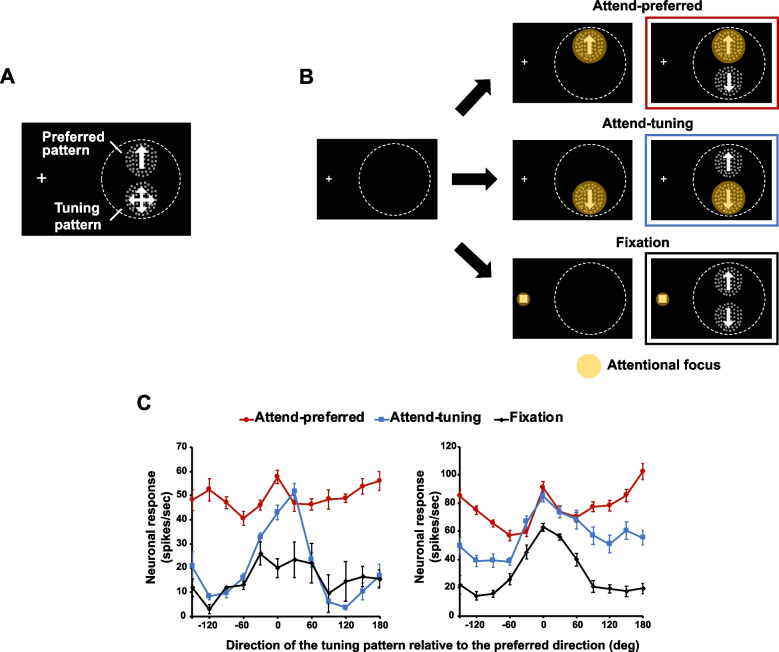
**A** Stimulus configuration of Experiment 1. The preferred pattern (always moving in the neuron’s preferred direction) and the tuning pattern (moving in one of 12 directions) were presented within a neuron’s RF. The fixation point was presented on the left side of the display. **B** Three attentional conditions. While an animal foveated the fixation point, it was cued to attend to either the preferred (attend-preferred) or tuning (attend-tuning) pattern and reported a directional change of the attended pattern. In the fixation condition, the animal was cued to the fixation point and had to report a change in the color of a small square superimposed on the fixation point while ignoring the RDPs. The yellow spot indicates the allocation of attention in each condition. **C** Responses of two single neurons in different attentional conditions. The abscissa represents the direction of the tuning pattern as a function of the distance to the preferred direction and the ordinate represents the response in spikes/second. Error bars indicate the standard error of the mean (SEM)

Two macaque monkeys were trained to selectively attend to a cued stimulus, while keeping gaze on a fixation point (Fig. 1A). We positioned two RDPs within a neuron’s RF, one RDP always moved in the neuron’s preferred direction (preferred pattern) and the other could move in one of 12 different directions from trial to trial (tuning pattern, in steps of 30°). The animals attended to either the preferred pattern (attend-preferred condition) or tuning pattern (attend-tuning condition) (Fig. 1B). A direction change could occur on one of the two patterns and the animals reported this change only when it happened on the attended pattern. In the fixation condition, they detected a color change of the fixation point while ignoring both RDPs. Animal F and M achieved 86 and 87% of change detection accuracy, respectively, indicating that they correctly perform the task.

Seventy-eight neurons were included in the analysis. Two examples of neuronal responses in different attentional conditions are shown in Fig. 1C. Responses in the attend-preferred condition (labeled as zero in the abscissa of Fig. 1C) were in general stronger than in the other conditions. The attend-preferred curve (red) is predominantly above the fixation (black) and attend-tuning (blue curves). Importantly the attend-preferred curve shows a non-Gaussian profile with a peak at the preferred direction and a dip at directions away from the preferred by 60–90°. This was particularly noticeable for the cell on the right. Thus, the tuning curve corresponding to the attend-preferred condition underwent a non-linear transformation when the animal directed attention to the preferred direction and ignored the tuning pattern. This transformation resembles a ring-of-inhibition around the attended-preferred feature.

**Fig. 2:**
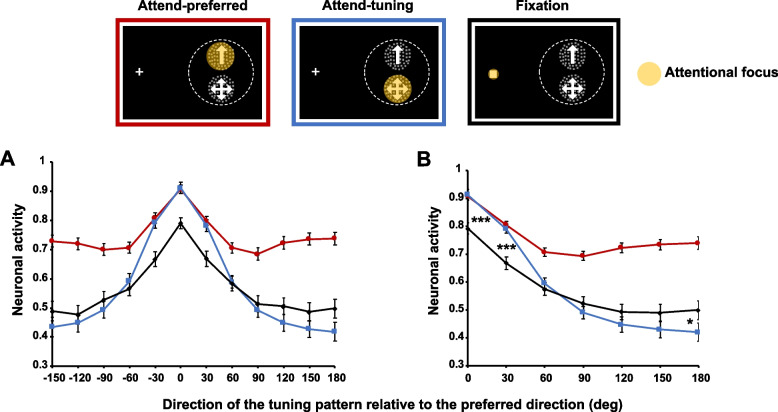
**A** Average normalized neuronal response in each attentional condition. **B** Normalized neuronal responses averaged across the same (absolute) directional difference (*n* = 78). Responses in the attend-tuning and the fixation conditions monotonically decrease as the offset between the preferred and tuning patterns increases. Responses in the attend-tuning condition are larger than that in the fixation condition when the direction of the tuning pattern is close to the neuron’s preferred direction (0 ~ 30°). This relationship reverses when the direction of the tuning pattern approaches the neuron’s anti-preferred direction. In the attend-preferred condition, however, responses are lowest when the direction of the tuning pattern is approximately 90° away from the preferred direction. Then, the responses increase as the directional difference increases. Error bars indicate SEM across the normalized responses of each neuron. * *p* < .05, *** *p* < .001

In order to determine the difference in responses between conditions, we normalized response of each neuron to the maximal response and then averaged this normalized response across neurons in each condition. We arranged the responses along the *x*-axis as a function of the difference between the preferred direction and the direction of the tuning pattern in a symmetrical manner to produce a response profile (Fig. 2A).

A repeated-measures analysis of variance (ANOVA) showed the main effect of attentional conditions that the neuronal response was significantly modulated by different attentional conditions (*F*(2, 122) = 47.058, *p* < 0.001). Subsequent pairwise comparisons showed that there was an increased overall response in the attend-preferred condition relative to the other conditions (all *p*s < 0.001 with Bonferroni correction). No significant difference was observed between the attend-tuning and the fixation conditions (*p* = 1). The direction of the tuning pattern relative to the preferred direction also significantly modulated neuronal responses (*F*(11, 671) = 45.768, *p* < 0.001), with the greatest response when the tuning pattern moved in the preferred direction (0° difference, all *p*s < 0.001) and a gradual decrease as the tuning pattern’s direction deviated from the preferred direction. The interaction between the attentional conditions and the directional difference was significant (*F*(22, 1342) = 13.561, *p* < 0.001). To elucidate the nature of this interaction, we examined the profile of attentional modulation under different attentional conditions. Because the response functions show a symmetric profile, we collapsed neuronal responses when the absolute directional difference between the preferred and the tuning patterns was the same (e.g., ± 30°) (Fig. 2B).

Neuronal response in the fixation condition peaked when the tuning pattern moved in the neuron’s preferred direction likely because both RDPs moved in the preferred direction and neither pattern extended into the RF’s inhibitory surround. This was assessed during initial mapping of the RF (see “Recordings” in “Methods”). The response reached its minimum when the tuning pattern moved in the neuron’s anti-preferred direction. There was a monotonic decrease in response as a function of the difference between the neuron’s preferred direction and the direction of the tuning pattern (Fig. 2B).

Neuronal response in the attend-tuning condition also monotonically decreased as the directions between the preferred and the tuning patterns became dissimilar. In this condition, neuronal responses were greater than those in the fixation condition when the direction of the tuning pattern was closer to the neuron’s preferred direction (at 0 and 30° differences, all *p*s < 0.001). On the other hand, responses in the attend-tuning condition were lower than those in the fixation condition when the tuning pattern moved in the neuron’s anti-preferred direction (mean difference (*M*_diff_) =  − 0.07, standard error (SE) = 0.04, *t*(76) = -2.101, *p* = 0.039). This effect is similar to feature-similarity gain modulation described in other studies [4].

In the attend-preferred condition, the maximal neuronal response was also observed when the directions of the preferred and tuning patterns were the same. However, we did not observe a monotonic response decrease with the direction of the tuning pattern relative to the preferred direction. Responses were lowest when the tuning pattern moved in directions approximately 90° away from the preferred direction, not when the tuning pattern moved in the anti-preferred direction. A repeated-measures ANOVA showed that average normalized neuronal response significantly changed depending on directional differences between the two RDPs (*F*(6, 450) = 28.367, *p* < 0.001). We conducted the Benjamini–Hochberg procedure to adjust multiple comparisons (FDR = 0.05, [28]). This procedure corrects multiple comparisons that are partially related, so it is more appropriate than Bonferroni correction which assumes independence of (categorical) data. We compared the minimum neuronal response at 90° difference to the responses at the other six directional differences. Responses at 0° difference (*M*_diff_ =  − 0.22, SE = 0.02, *p* < 0.001, BH critical value = 0.008) and at 30° difference (*M*_diff_ =  − 0.11, SE = 0.02, *p* < 0.001, BH critical value = 0.008) were significantly greater than the minimum neuronal response. The response at 60° difference did not significantly differ from the response at 90° difference (*M*_diff_ =  − 0.01, SE = 0.01, *p* = 0.159, BH critical value = 0.008). When the directional difference between the two RDPs became larger, neuronal responses were much greater than the minimum response (90 vs. 120°: *M*_diff_ = 0.03, SE = 0.01, *p* = 0.007, BH critical value = 0.025; 90 vs. 150°: *M*_diff_ = 0.04, SE = 0.02, *p* = 0.011, BH critical value = 0.033; 90 vs. 180: *M*_diff_ = 0.05, SE = 0.02, *p* = 0.026, BH critical value = 0.05).

In addition, we looked into at which directional difference individual neurons showed minimum response (i.e., maximum suppression) to estimate the center of a suppressive surround. We found that the minimum responses were mostly observed when the directional difference was around 60 ~ 90° (see Additional file 1). This result indicates that the attend-preferred condition causes a suppressive surround for motion quasi-orthogonal to a neuron’s preferred direction.

### Tuning curves

Neuron’s tuning curves have been modeled using Gaussian functions [8]. However, because responses do not clearly follow a monotonic profile in the attend-preferred condition, we fitted two different models to the average normalized neuronal response (population response) in each experimental condition, a single Gaussian and a sum of two Gaussians. A single Gaussian model was:

$$f\left(x\right)=b+g{*e}^{-\frac{{(x-\mu )}^{2}}{2{\sigma }^{2}}}$$

where *b* is the baseline, $g$ is the response gain, $\mu$ is the center, and $\sigma$ is the width of the Gaussian. A sum of two Gaussians model was the combination of two regular Gaussian functions:

$$f\left(x\right)=b+{g}_{1}{*e}^{-\frac{{(x-\mu )}^{2}}{2{{\sigma }_{1}}^{2}}}+ {g}_{2}{*e}^{-\frac{{(x-\mu )}^{2}}{2{{\sigma }_{2}}^{2}}}$$

**Fig. 3:**
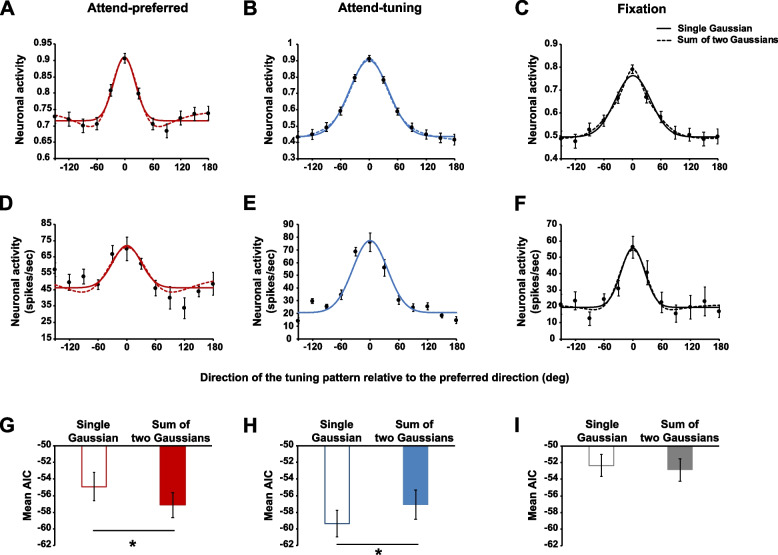
**A–C** Different model fits to the average normalized neuronal response (population level) in the attend-preferred, attend-tuning, and fixation conditions (*n* = 78). Solid curve indicates the single Gaussian model fit and dashed curve indicates the sum of two Gaussians model fit (see **D–F** for an example neuron). **G–I** Akaike Information Criterion (AIC) for each model fit was measured at individual neuron level. Smaller AIC value means better model fit (*n* = 78). Outlined and filled bars indicate the mean AIC of the single Gaussian and the sum of two Gaussians models, respectively. Error bars indicate SEM. * *p* < .05

The two Gaussian functions were center aligned (sharing the same *µ* parameter). In the attend-preferred condition (Fig. 3A), the sum of two Gaussians (adjusted *R*^2^ = 0.990) model explained the population response better than the single Gaussian model (adjusted *R*^2^ = 0.886). This was mainly due to the ability of the sum of two Gaussians (one Gaussian having a positive gain and narrower width than the second Gaussian with negative gain and larger width) to account for the decrease in response in the vicinity of the preferred direction relative to directions farther away from the preferred one. This effect resembles the feature-based surround suppression reported in previous behavioral and modeling studies [15–22]. In the other attentional conditions (Fig. 3B, C), both models could explain the response profiles equally well (all adjusted *R*^2^, attend-tuning condition: single Gaussian (0.994), sum of two Gaussians (0.999); fixation condition: single Gaussian (0.957), sum of two Gaussians (0.987)).

In order to quantify these observations, we compared model fits at the individual neuron level. Our curve fitting algorithm failed to fit a given individual neuronal response if the fit did not converge due to variability in the data, or because some data points were missing (maximum number of iterations allowed = 400). We included individual neurons in the analysis only if both models successfully fit their responses. We then compared different model fits to the same neuronal response (i.e., pairwise comparison): 69 neurons in the attend-preferred condition, 53 neurons in the attend-tuning condition, and 59 neurons in the fixation condition were included in the analysis. Example model fits to normalized responses are shown in Fig. 3D–F. As a goodness-of-fit measure, we computed the Akaike Information Criteria (AIC) for each model fit. The AIC takes into account the number of parameters in each model, which is lower in the single Gaussian compared to the sum of two Gaussians. In the attend-preferred condition (Fig. 3G), the AIC was greater for the single Gaussian than for the sum of two Gaussians (*M*_diff_ = 2.22, SE = 1.03, *t*(68) = 2.148, *p* = 0.035), meaning that the latter model explained the data better, considering the number of parameters. Conversely, the single Gaussian fitted the data better in the attend-tuning condition (Fig. 3H), demonstrating a smaller AIC value than the sum of two Gaussians (*M*_diff_ =  − 2.25, SE = 0.92, *t*(52) =  − 2.448, *p* = 0.018). In the fixation condition (Fig. 3I), there was no significant difference in AIC between the two models (*M*_diff_ = 0.53, SE = 0.88, *t*(58) = 0.605, *p* = 0.548).

Overall, for the population as well as for single neurons the sum of two Gaussians model fits the data better only in the attend-preferred condition. In the other conditions, either both models fit the data equally well (fixation) or the single Gaussian model performs better than the sum of two Gaussians model (attend-tuning). These results indicate that feature-based surround suppression became evident only in the attend-preferred condition.

### Modeling feature-based surround suppression

**Fig. 4:**
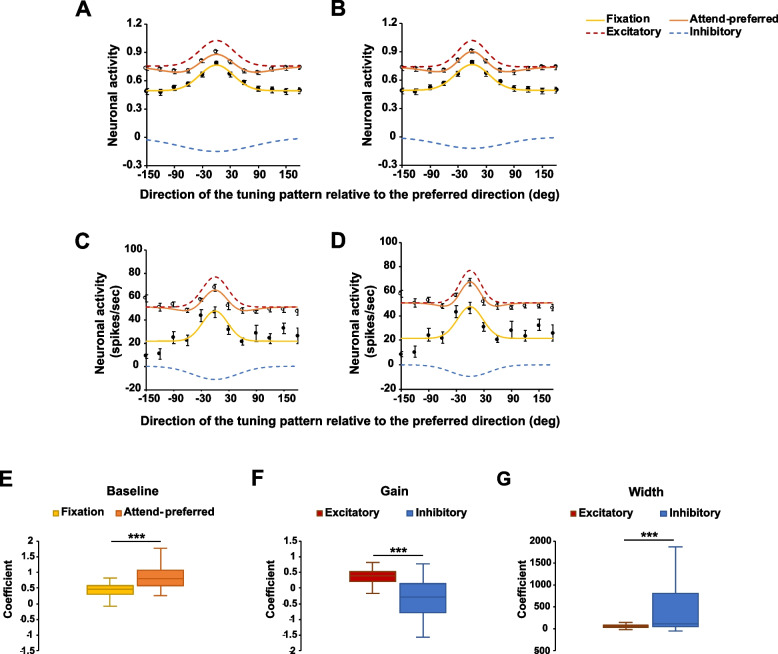
**A,B** Model fits to the averaged normalized neuronal responses (population level, *n* = 78). Outlined circles and filled circles indicate the data in the attend-preferred condition and in the fixation condition, respectively. **A** For the sum of two Gaussians model in the attend-preferred condition, coefficients of the excitatory Gaussian (gain and width) were the same as those of the single Gaussian model in the fixation condition. **B** All coefficients of the sum of two Gaussians model were free to vary. **C,D** Examples of the model fits to individual neuronal responses. In **C**, the coefficients of the excitatory Gaussian were constrained while they were unconstrained in **D**. **E–G** Median coefficients of the model fits to the normalized individual neuronal data (*n* = 69). **E** Baseline coefficients when the single Gaussian model fit the data in the fixation condition and when the sum of two Gaussians model fit the data in the attend-preferred condition. **F** Gain and **G** width coefficients of the excitatory and inhibitory Gaussians when the sum of two Gaussians model fit the data in the attend-preferred condition. Note that the gain coefficients of the excitatory Gaussian were the same as those of the single Gaussian fits in the fixation condition. Error bars indicate SEM. *** *p* < . 001

The attend-preferred condition may have allowed us to isolate the feature-based surround suppression effect because attention was always on the same feature (preferred direction) while the distractor tuning pattern changed direction from trial to trial. Thus, keeping attention on the preferred direction reveals the surround suppressive effect on distracting features. One possible explanation for this effect is the interaction between excitatory- and inhibitory-tuned inputs into a neuron during the allocation of feature-based attention. In order to model this interaction, we modeled the excitatory and inhibitory fields of direction-selective neurons as two Gaussian functions with positive and negative gain, respectively. We first fit the average normalized neuronal responses in the fixation condition with the single Gaussian model. The same model for the neurons’ tuning curves was used here (see the “Tuning curves” section above), except that the parameter for the width of the Gaussian was $\sigma$, rather than 2 $\sigma$ for simplicity and that the center of the Gaussian $\mu$ was always 0 because neuronal response was maximized when the preferred and tuning pattern moved in the same direction (no directional difference). An important point here is that we assumed that in the fixation condition, the predominant contribution to the response is provided by excitatory-tuned inputs into the cell with $\sigma$ representing the width or selectivity of the inputs. The contribution of inhibitory inputs into the cell is considered relatively small here than when attention is directed to the RDPs within a RF, and will be captured by the single Gaussian [29]. We cannot exclude the possibility of inhibition during fixation. However, this inhibition would be relatively small in magnitude and not related to feature selectivity (i.e., motion direction), thus it would be difficult to measure in the present experiment. The fitting result is illustrated in Fig. 4A (yellow line). The coefficients and goodness-of-fit measures are shown in Table 1.

**Table 1:** Coefficients of the model fits to the average normalized neuronal responses (population level, *n* = 78) in the fixation and attend-preferred conditions and goodness-of-fit measures

		Fixation	Attend-preferred (constrained)	Attend-preferred (unconstrained)
Baseline		0.50	0.76	0.74
1st Gaussian (excitatory)	Gain	0.27	0.27	0.28
	Width	51.29	51.29	42.65
2nd Gaussian (inhibitory)	Gain	N/A	− 0.15	− 0.12
	Width		110.82	96.94
Goodness-of-fit	SSE	0.002731	0.001433	0.0003139
	*R*^2^	0.9738	0.9667	0.9927
	Adjusted *R*^2^	0.9679	0.9594	0.9886
	RMSE	0.01742	0.01262	0.006696

Second, we fit the average normalized neuronal response in the attend-preferred condition with a sum of two Gaussians model. We “set” the parameters of one Gaussian to the same parameters obtained from the fixation condition under the assumption it approximates the excitatory field provided by the tuned inputs into the cell. For the second Gaussian, the coefficients were not constrained. We assumed that this second Gaussian would have negative gain representing inhibitory-tuned inputs into the neuron recruited (or magnified) by feature-based attention. The equation is:

$$f\left(x\right)=b+(0.2713{\times e}^{-{\left(\frac{x-\mu }{51.29}\right)}^{2}})+({g}_{i}{\times e}^{-{\left(\frac{x-{\mu }_{i}}{{\sigma }_{i}}\right)}^{2}})$$

The parameters *b* (baseline), ${g}_{i}$ (gain), and ${\sigma }_{i}$ (width) were free to vary (while *μ*_*i*_ was set to 0).The baseline was not fixed since it may capture overall changes in response due to spatial attention or arousal, i.e., in the fixation condition attention was directed to the fixation point while in the attend-preferred condition attention was directed to the RF likely producing a response increase for all directions [8]. The other parameters, ${g}_{i}$ and ${\sigma }_{i}$, represent the gain and width of the inhibitory inputs into a neuron. Negative values of ${g}_{i}$ would reflect increase in the gain of inhibitory inputs/field by attention.

In the attend-preferred condition, the baseline of neuronal response increased (red dashed line) relative to the baseline in the fixation condition (yellow line) (Fig. 4A and Table 1). This may reflect the increase in responses due to directing attention into the RF. As anticipated, the second Gaussian which represents the inhibitory field (blue dashed line) showed a negative gain (${g}_{i}$ < 0) and a broader (${\sigma }_{i}>\sigma$) width than the first Gaussian representing the excitatory field (51.29 vs. 110.8, Table 1). Notice the excitatory field was estimated from the Gaussian fit in the fixation condition, where we assume the contribution of the inhibitory field was small relative to that of the excitatory field. We repeated the fitting procedure but letting the coefficients of both Gaussian functions freely vary (Fig. 4B). The improvement in the goodness-of-fit of the model and changes in the coefficients were negligible (Table 1), indicating that changes induced by feature-based attention on the inhibitory field can account for the changes in tuning profile.

We repeated a similar procedure at the level of single neurons. We first fit the single Gaussian model to the normalized individual neuronal responses in the fixation condition and used the coefficients to model the neuron’s excitatory field in the attend-preferred condition. The coefficients for the second Gaussian were not constrained. Neurons were excluded from the analyses if the fitting procedure was not successful due to missing data points or severe variability in the data (i.e., fit did not converge). Consequently, 69 neurons were included in the analysis. The median baseline, gain, and width coefficients of the single Gaussian model fit in the fixation condition were 0.4656, 0.3996, and 54.83, respectively. In the attend-preferred condition, after constraining the first (excitatory) Gaussian coefficients, the median baseline was 0.808 and the median gain and width of the second (inhibitory) Gaussian were − 0.2889 and 113.97, respectively. Wilcoxon signed-rank tests showed that the baseline was significantly elevated in the attend-preferred condition (*Z* = 4.1105, *p* < 0.001, Fig. 4E). The inhibitory fields had significantly lower gains (*Z* =  − 3.9371, *p* < 0.001, Fig. 4F) and the broader widths (*Z* = 5.0612, *p* < 0.001, Fig. 4G) than the excitatory fields. This shows that model fits to the individual neuron data show the same results as in the population-level analysis.

### Behavioral effects of feature-based surround suppression

**Fig. 5:**
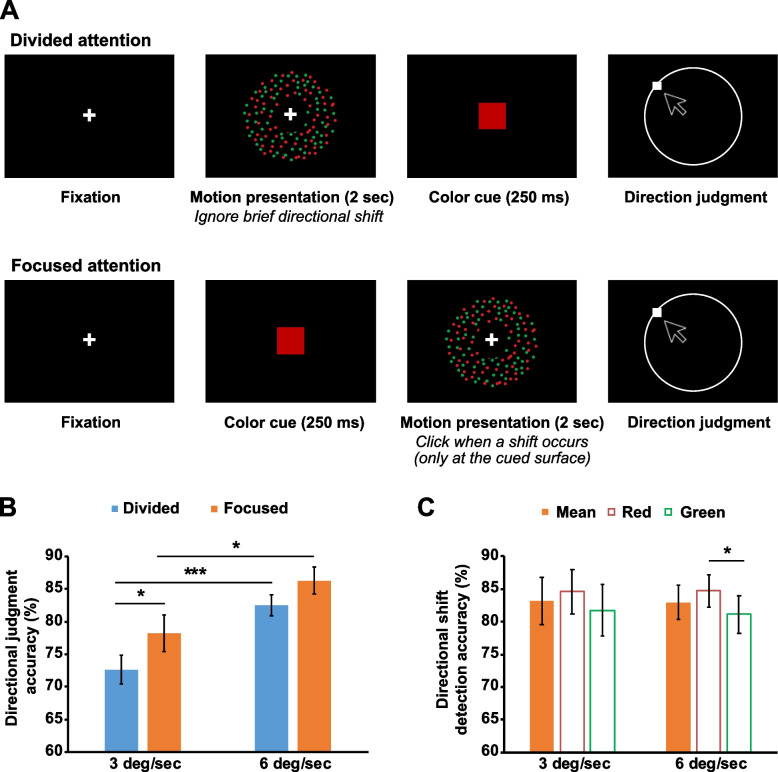
**A** Experimental conditions and procedure of human behavioral experiment. In the divided attention condition, participants attended to both RDPs equally, ignoring any directional shift. A color cue was presented after RDPs disappeared, and then, participants reported a direction of the cued RDP by clicking along a white circular outline. In the focused attention condition, a color cue indicated which RDP participants had to attend. Participants were asked to detect a directional shift on the cued RDP if it occurred, and then reported motion directions of this RDP as in the divided attention condition. **B** Direction judgment was more accurate in the focused attention condition, and when motion speed was faster (*n* = 14). **C** Detection of directional shifts (only in the focused attention condition) was not affected by motion speed. However, direction discrimination was more accurate when the attended RDP was red. Error bars indicate SEM. * *p* < .05, *** *p* < . 001

We investigated the effect of feature-based surround suppression on behavior using motion repulsion, subject to feature-based attentional modulation [26, 27]. Motion repulsion was measured when participants divided their attention to two superimposed RDPs (divided attention condition) or focused on one of them (focused attention condition, Fig. 5A). The superimposed RDPs were separated by different colors and their directional offsets systematically varied (10 to 50°). The speed of motion for the RDPs could be 3°/s or 6°/s.

First, we analyzed how feature-based attention and motion speed influenced participants’ direction judgment accuracy—i.e., whether reported directions fell within a valid response range (Fig. 5B, see “Data analysis” in “Methods”). Direction judgment accuracy in the focused attention condition was measured only if the attention task (detecting a brief directional shift) was successfully performed. A repeated-measures ANOVA demonstrated significant main effects of the attentional conditions (*F*(1, 13) = 6.683, *p* = 0.023) and motion speed (*F*(1, 13) = 22.443, *p* < 0.001) on direction judgment accuracy. Post hoc pairwise comparisons with the Bonferroni correction showed that direction judgment was more accurate in the focused attention condition than in the divided attention condition (*M*_diff_ = 4.59%, SE = 1.78%) for both motion speeds (3°/s: *p* = 0.044, 6°/s: *p* = 0.065 (trend)) because participants tracked only one motion direction in the focused attention condition. Direction judgment accuracy was also higher when motion speed was faster (*M*_diff_ = 9.03%, SE = 1.91%) in both attentional conditions (divided: *p* < 0.001, focused: *p* = 0.01). Color of the target RDP did not influence direction judgment (*F*(1, 13) = 0.051, *p* = 0.825). All interactions between the variables were not significant.

Figure 5C shows the mean accuracy of directional shift detection in the focused attention condition. The mean accuracy was 83.14% (SD 13.45%) and 82.93% (SD 9.79%) when motion speed was 3°/s and 6°/s, respectively. They did not statistically differ (*p* = 0.915), indicating that difficulty of the attention task was well controlled across different speed conditions. When it was broken down by the color of the attended RDP (target), the main effect of the target surface color on directional shift detection was significant (*F*(1, 13) = 13.995, *p* = 0.002). Detecting directional shifts was better when the target RDP was red than when it was green (*M*_diff_ = 3.21%, SE = 0.86%) for both motion speeds (3°/s: *p* = 0.062 (trend), 6°/s: *p* = 0.015). The performance advantage in the red RDPs likely represents strong attentional guidance of red [30, 31]. Interaction between motion speed and the color of the target RDP was not significant (*F*(1, 13) = 0.12, *p* = 0.734).

**Fig. 6:**
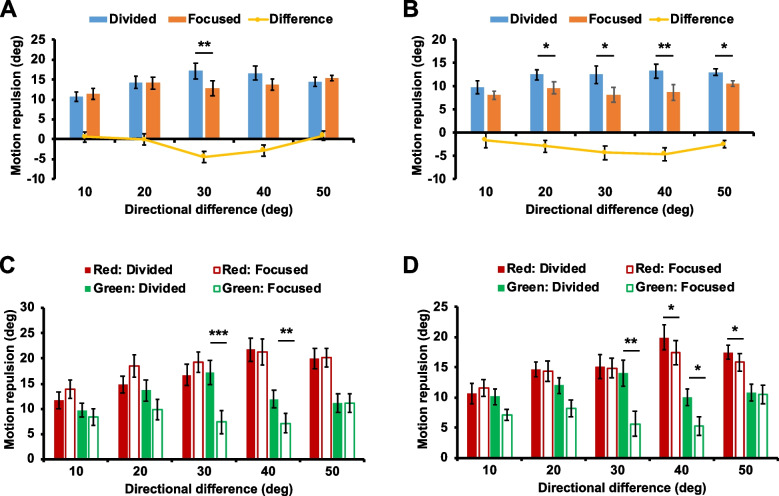
**A** When motion speed was 3°/s, the amount of motion repulsion between the two attentional conditions significantly differed when the directional difference was 30° (*n* = 14). The same effect was marginal when the directional difference was 40° (*p* = .056). **B** When motion speed was 6°/s, motion repulsion was reduced in the focused attention condition and it was true across all directional differences, except at 10° difference. We further broke down the data by the color of the target RDP. **C** When motion speed was 3°/s, attentional modulation on motion repulsion was evident only if the target RDP was green. **D** On the other hand, when motion speed became faster, attentional modulation on motion repulsion was observed regardless of the color of the target RDP. Error bars indicate SEM. * *p* < .05, ** *p* < .01, *** *p* < .001

Our main interest was examining feature-based surround suppression reflected in motion repulsion. To address this, we analyzed motion repulsion of the trials in which participants correctly discriminated motion direction. The main effect of motion speed significantly affected motion repulsion (*F*(1, 13) = 54.343, *p* < 0.001): faster motion speed (6°/s) reduced motion repulsion (*M*_diff_ = 3.47°, SE = 0.47°) as previously reported [32–34]. When motion speed was 3°/s (Fig. 6A), the main effect of the attentional condition on motion repulsion was not significant (*F*(1, 13) = 2.224, *p* = 0.16), whereas motion repulsion significantly varied depending on the directional difference between the two RDPs (*F*(4, 52) = 2.715, *p* = 0.04). Importantly, motion repulsion was influenced by the interaction between the attentional condition and the directional difference (*F*(4, 52) = 3.958, *p* = 0.007). Motion repulsion was significantly reduced in the focused attention condition when the directional difference was 30° (*M*_diff_ = 4.40°, SE = 1.41°, *p* = 0.008) and this effect was marginal when the directional difference was 40° (*M*_diff_ = 2.81°, SE = 1.34°, *p* = 0.056). We also compared the suppressive effect, defined by the difference in motion repulsion between the two attention conditions (focused vs. divided), at 30° difference (where the reduction of motion repulsion was apparent) with the effects at the other directional differences. Pairwise comparisons with the Benjamini and Hochberg procedure (FDR = 0.05) showed that the suppressive effect at 30° difference was significantly greater than the effects at 10° (*M*_diff_ = 5.06°, SE = 2.01°, *p* = 0.026, BH critical value = 0.038) and 20° differences (*M*_diff_ = 4.34°, SE = 1.64°, *p* = 0.02, BH critical value = 0.025), and it tended to be greater than the effect at 50° difference (*M*_diff_ = 5.35°, SE = 1.95°. *p* = 0.017, BH critical value = 0.013). To provide converging evidence, we fit a quadratic model to the data as an approximation of the surround suppression [35] and compared its goodness-of-fit with that of a linear model. Whereas the linear model did not properly fit the data (adjusted *R*^2^ =  − 0.305, AIC = 11.344), the quadratic model, an approximation of the surround suppression [35], showed a better fit (adjusted *R*^2^ = 0.734, AIC = 6.826). Reduction of motion repulsion at around 30 ~ 40° difference suggests that feature-based surround suppression played a role by inhibiting an unattended motion direction that was near the attended motion direction.

When motion speed was 6°/s (Fig. 6B), the attentional condition significantly modulated motion repulsion (*F*(1, 13) = 19.557, *p* = 0.001), demonstrating that motion repulsion was generally smaller in the focused attention condition (*M*_diff_ = 3.18°, SE = 0.72°). Directional difference (*F*(4, 52) = 1.652,* p* = 0.175) and the interaction between the attentional condition and the directional difference (*F*(4, 52) = 1.049, *p* = 0.391) did not significantly affect motion repulsion. Motion repulsion was significantly smaller in the focused attention condition at all directional differences, except at 10° difference, and the difference in motion repulsion did not statistically vary across directional differences. Therefore, unlike when motion speed was 3°/s, it was difficult to specify the effect of feature-based surround suppression.

We further broke down the data by the color of the target RDP to see how this factor is associated with motion repulsion. When motion direction was 3°/s, the amount of motion repulsion was different depending on the color of the target RDP (*F*(1, 13) = 28.589, *p* < 0.001; Fig. 6C). Motion repulsion was smaller when the target RDP was green than when it was red (*M*_diff_ = 7.07°, SE = 1.32°). Although the attentional condition did not affect motion repulsion (*F*(1, 13) = 2.07, *p* = 0.174), there was a significant interaction between the target RDP color and the attentional condition (*F*(1, 13) = 9.199, *p* = 0.01). We conducted an ANOVA separately for each target RDP color to investigate this interaction in more detail. When the target RDP was red, motion repulsion was not affected by the attentional conditions (*F*(1, 13) = 1.188, *p* = 0.296) but by the directional difference between the two RDPs (*F*(4, 52) = 7.522, *p* < 0.001). The interaction between these two variables was not significant (*F*(4, 52) = 0.8, *p* = 0.531). Hence, there was no evidence for feature-based surround suppression. On the other hand, when the target RDP was green, the main effect of the attentional conditions was significant (*F*(1, 13) = 22.631, *p* < 0.001), indicating that feature-based attention reduced motion repulsion (*M*_diff_ = 3.96°, SE = 0.83°) as previously reported [26]. Motion repulsion was not modulated by the directional difference (*F*(4, 52) = 0.802, *p* = 0.529), but the interaction between the attentional conditions and directional difference was significant (*F*(4, 52) = 6.699, *p* < 0.001). Motion repulsion significantly decreased in the focused attention condition when the directional difference was around at 20 ~ 40° (20°: *p* = 0.051 (marginal), 30°: *p* < 0.001, and 40°: *p* = 0.005). This indicates that feature-based surround suppression in the 3°/s condition was mainly derived from the trials where the target RDP was green.

When motion speed was 6°/s, motion repulsion was modulated by the color of the target RDP (*F*(1, 13) = 14.491, *p* = 0.002), showing smaller motion repulsion in the green RDPs (*M*_diff_ = 4.72°, SE = 1.24°; Fig. 6D). In addition, the attentional condition significantly affected motion repulsion (*F*(1, 13) = 16.507, *p* = 0.001). Motion repulsion was smaller in the focused attention condition than in the divided attention condition (*M*_diff_ = 3.07°, SE = 0.76°). The interaction between the target RDP color and the attentional condition was not significant (*F*(1, 13) = 0.865, *p* = 0.369). Unlike when motion speed was 3°/s, the attentional condition modulated motion repulsion even when the color of the target RDP was red (*F*(1, 13) = 5.177, *p* = 0.04). Post hoc pairwise comparisons showed that motion repulsion was reduced in the focused attention condition (*M*_diff_ = 2.22°, SE = 0.97°) and it was significant when the directions of RDPs differed by 40 ~ 50° (40°: *p* = 0.036, 50°: *p* = 0.02). When the target RDP was green, feature-based attention also significantly reduced motion repulsion (*F*(1, 13) = 8.148, *p* = 0.014; focused vs. divided: *M*_diff_ =  − 3.93°, SE = 1.38°). The reduction was at the trend level at 10 ~ 20° difference (10°: *p* = 0.053, 20°: *p* = 0.085) and became significant at 30 ~ 40° difference (30°: *p* = 0.008, 40°: *p* = 0.021). Motion repulsion patterns for green RDPs were consistent across different motion speeds, whereas feature-based attention played a role only if motion speed was faster when the target RDP was red. Therefore, the almost universal decrement in motion repulsion in the 6°/s condition might have resulted from the interaction between the color of the target RDP and motion speed.

## Discussion

Our neurophysiological findings in monkey show that attention to a neuron’s preferred motion direction modulates its direction tuning curve, imposing a suppressive surround around the attended direction. In addition, our behavioral study demonstrates feature-based surround suppression on motion repulsion in humans. We modeled this non-linear effect of feature-based attention as differential gain modulations of widely tuned inhibitory inputs and narrowly tuned excitatory inputs into neurons.

### Effects of feature-based attention on motion direction tuning curves

Feature-based attention allows enhancing the processing of attended features while suppressing the processing of unattended ones [5]. These effects of feature-based attention have been captured by the feature-similarity gain model, which proposes that modulation of a neuron’s response is a monotonic function of the differences between the neuron’s preferred feature and the attended feature [4, 8]. Feature-based attention produces a maximal response enhancement for the attended feature and a progressive decrease of responses that becomes suppression relative to a neutral condition as the attended feature deviates from the preferred feature of the neurons. Our results in this study do not match those reported by previous electrophysiological studies of feature-based attention in macaques [4, 8]. Here, we found that the decay in the intensity of the feature-based attention response enhancement was not monotonic but reached its minimum in the vicinity of the attended feature producing a non-linearity in the tuning curve that cannot be directly explained by a gain modulation.

One possible explanation for the difference between our results and those of the previous study is that in [4] there was a large separation between target and distractor; therefore, the feature-based attentional response modulation may not have been sufficiently large to reveal the precise shape of the feature-similarity modulation. Location-based surround suppression predicted by the Selective Tuning model was formulated for stimulus conditions in which distractors are adjacent to the attended stimulus and attention filters out their contribution to the neuron’s response. In our study the attended stimulus and distractor were nearby, within the same RF, resembling the formulation in the Selective Tuning model. Feature-based attentional modulation was stronger because the proximity of the RDPs may have triggered stronger inhibitory circuit dynamics. Indeed, attentional modulation is stronger when targets and distractors are positioned inside the RF relative to when they are positioned one inside and the other outside the RF [8, 36]. Moreover, distractor interference is stronger when both stimuli are within the same hemifield relative to when they are in different hemifields [37]. Additionally, in our study, we maintained the preferred pattern in the RF, which may have strongly driven neuronal responses [38] in a way that the modulation was detectable with a reasonable sample of recorded neurons and trials. Supporting the later view, we observed a variability in the intensity of the non-linear effect across neurons (Fig. 1C). This interpretation suggests that feature-based attention might interact with spatial attention (spatial attention refers to the allocation of attention to spatial locations, while feature-based attention refers to the allocation of attention to different features independently of their spatial locations). Our previous study provided supporting evidence for the interaction between spatial and feature-based surround suppression [35]. We showed that the suppressive effect is greatest when the two (spatially) adjacent targets are also close in feature space. However, we acknowledge that this topic is still a matter of debate [39–42] and further research is required to elucidate the nature of feature-based attention.

Since the preferred pattern was constantly presented within the RF across different attention conditions, pure motion sensory adaptation to this stimulus is unlikely to explain our results [43, 44]. However, the effects of adaptation might have interacted with attention because attention varied along the different combinations of RDPs in the attend-preferred and attend-tuning conditions (c.f., adaptation to different cues [12]).

### Modeling the response modulation

Models of MT neurons have proposed that feature tuning is a function of excitatory-tuned inputs from upstream neurons and inhibitory inputs from neurons within the same area. This idea is captured by normalization models [45], which propose that inhibitory inputs into a neuron are not tuned for stimulus features. Normalization models can account for observed modulations of neuronal responses by attention [46]. Attention could modulate inputs into neurons, and because the same inputs activate the normalization pool in different manners depending on the size of the attentional focus, a variety of effects could be achieved at the level of single-cell responses [45]. Others have further proposed tuned normalization as a mechanism to explain attentional modulation across neurons [47, 48].

One possibility that explains our results is a tuned normalization pool modulated by feedback signals from high-order areas into MT/MST. However, for a gain feedback modulation to explain the feature-based surround suppression, the tuning of the feedback modulation, or of the inhibitory neuronal pools they activate (directly or indirectly) needs to be wider than the tuning of feedforward excitatory inputs. It is possible the feedback does not directly drive inhibitory neurons but excitatory cells that in turn activate the inhibitory pool. It is difficult with the available data to provide a detailed circuit layout. But we can elaborate on a hypothesis as follows: (1) the tuning/width of the inhibitory inputs into a neuron are wider than those of excitatory inputs, and (2) attention modulates the gain of inhibitory inputs more strongly than excitatory inputs. The appeal of this proposal relies on how gain changes differentially applied to MT/MST excitatory and inhibitory fields could produce a non-monotonic non-linear modulation when the modulated excitatory and inhibitory fields are integrated. We should note that feedforward inputs into a neuron could increase their strength with attention as long as the effect is smaller than the one of feedback inputs. Indeed, this may be the case according to studies isolating the modulation of responses in area V1 [36]. If feedback gain signals were to originate downstream from the recorded area, and given that the attentional modulation of responses grows along the hierarchy of visual processing [49, 50], it is reasonable to assume that within a given area changes in the strength of feedback signals would be greater than changes in the strength of feedforward inputs. This assumption, however, needs to be further tested.

Previous studies have reported that the tuning of inhibitory neurons for stimulus features is wider than the tuning of excitatory cells [51]. In monkey dorsal MST, narrow-spiking putative interneurons are more broadly direction tuned than broad-spiking putative pyramidal cells [52]. Given that parvalbumin positive (PV) cells are the most abundant interneuron type in MT/MST [53], and they are involved in gain control of pyramidal cells [54], it could be that PV cells recruited by top-down inputs [55] provide the wide strong inhibitory drive that produces the non-linear change in the tuning curves.

Remarkably, a computational model, Selective Tuning model, predicts the effects described here as resulting from top-down feedback signals modulating the landscape of neuronal population activity in visual areas such as MT. Indeed, studies using moving RDPs have shown that the latency of the attentional effects on the responses of direction-selective neurons is shorter in the lateral prefrontal cortex than in MT [50], suggesting that top-down feature-based attentional signals originate in areas of the prefrontal cortex and feedback into visual cortex to modulate processing [55, 56]. Our results add detail to the way in which this specific modulation is implemented at the levels of single neurons and circuitry in MT that can be incorporated into models like the Selective Tuning model to generate detailed predictions at microcircuit level. One thing to note is that MST neurons have larger RFs than MT neurons that in many cases crossed the vertical meridian, and MST neurons are tuned for spiral motion. In addition, attentional effects are systematically stronger in MST than in MT [49]. Unfortunately, the sample we recorded from was small and we could not reliably analyze MST and MT neurons separately for this specific experiment.

### Behavioral effects of feature-based surround suppression

We observed that attention to one of the two superimposed RDPs reduced motion repulsion and this reduction was greatest when the RDPs moved in similar directions. This feature-based surround suppression effect varied depending on bottom-up factors such as motion speed and the color of the target RDP which indicates an interplay between top-down feature-based attention and bottom-up factors. The present results appear to be inconsistent with the previous report that the color of motion surfaces does not influence motion repulsion [57]. However, in their study, motion repulsion was not measured separately for the motion surface colors; hence, the effect of the surface color might have not been addressed. Greater motion repulsion in red surfaces suggests that these surfaces are more strongly affected by inhibition. Such a result is consistent with a recent study that showed red facilitated response inhibition compared to green [58]. It is also possible that different color wavelengths may influence motion repulsion. When the speed of the motion was faster, producing more signal strength, it is likely that the balance in mutual inhibition between the two-colored surfaces was more even, which then allowed for feature-based attentional modulation and surround suppression to be seen on the red surface.

One puzzling finding was that feature-based surround suppression in our behavioral experiment in humans was produced when the directional difference between two motions was smaller (30 ~ 40°) than in the neurophysiological experiment in monkeys (60 ~ 90°). The previous behavioral studies reported broader feature-based suppressive surrounds in the motion direction dimension than those in the other feature dimensions (maximum suppression around at around 90° difference, [20, 21]), a range similar to what we found in the neurophysiological experiment [59–61]. There are several possible explanations for the discrepancy between our two experiments. It may be that responses of MT single neurons tuned for motion direction translate into a different population response profile in areas downstream from MT/MST (e.g., lateral intraparietal area or the prefrontal cortex). In favor of this hypothesis, it has been reported that during a task that requires categorization of motion directions, lateral intraparietal and prefrontal neurons change their tuning while MT neurons do not [62]. It may also be that a narrower surround suppression profile in our behavioral experiment could be due to the nature of motion repulsion. Motion repulsion is typically attenuated as the two superimposed motions move in more dissimilar directions. In addition, human participants were asked to report motion directions with high precision, whereas monkeys had to detect directional changes while maintaining attention on an eccentric motion pattern. Finally, we used colored, superimposed RDPs in the behavioral experiment, while white, spatially separated RDPs in the neurophysiological experiment. Finally, biases idiosyncratic to each species (humans vs. monkeys) may have also influenced the profile of feature-based surround suppression.

The current behavioral paradigm allowed insight into feature-based surround suppression mechanisms. As motion repulsion is thought to occur due to mutual inhibition between competing pools of direction-selective neurons [63], it is possible increasing the gain of inhibitory relative to excitatory inputs into neurons within the focus of attention produces the observed effect, i.e., shifting the population tuning back towards the veridical direction from the repulsed one. Our experimental paradigm more directly demonstrates feature-based surround suppression of the Selective Tuning model relative to the previous ones.

## Conclusions

Our results demonstrated a consistent attentional surround suppressive effect at both neurophysiological and behavioral levels and resolved seemingly contradictory findings of feature-based attention: the feature-similarity gain modulation of neuronal inputs and tuning curves and the attentional impact on neural population responses predicted by the Selective Tuning model. We also modeled this suppressive effect, suggesting how non-linear changes in neural tuning and behavior can emerge from gain modulation induced by feature-based attention. Ultimately, our work provides a unified framework for the effects of feature-based attention on neuronal responses and behavior.

## Methods

### Neurophysiology in macaque monkeys

Research with non-human primates represents a small but indispensable component of neuroscience research. The researchers in this study are aware and are committed to the responsibility they have in ensuring the best possible science with the least possible harm to the animals [64].

#### Apparatus and stimuli

We recorded the responses of direction-selective neurons in areas MT and MST (*n* = 107) of two male adult macaque monkeys in different task conditions. After initial training, a head post, a scleral search coil [65] to monitor eye position [66], and a recording chamber were implanted in each animal. A custom computer program running on an Apple Macintosh PowerPC controlled the stimulus presentations, monitored eye position and behavioral responses during the experiments, and recorded the behavioral and neuronal data. The experiments reported in this study were conducted according to local and national rules and regulations and were approved by the Regierungspraesidium Tuebingen (Germany).

RDPs consisted of small bright dots with a density of 5 dots/deg^2^ within a stationary circular virtual aperture on a dark computer monitor. The luminance of the dots was 55 cd/m^2^ and the viewing distance was 57 cm. The size of the RDPs was adjusted (varied from about 1 to 3° in diameter) depending on the size of the recorded neuron’s RF so that both RDPs fell within the neuron’s classical RF. Eccentricity of the RFs’ centers from the fixation point varied between 3 and 10°, contralateral to the recorded hemisphere. Animals did not perform the task when RFs were more eccentric or when eye movements interfered with trial execution if RFs were too close to the fixation point. In every trial, we presented two RDPs of equal size at separate locations inside the neurons’ RF and their centers were approximately equally distant from the fixation point. The two locations within the RF were chosen in a manner that different directions of a single RDP elicited similar responses when presented at either location. One RDP always moved in the neuron’s preferred direction, which was estimated in a separate block of trials by online display of the responses to a single RDP inside the RF moving in different directions while the animal was fixating a dot at the screen center (see [8]). The other pattern could move in one of twelve different directions spaced every 30°. Movement of the dots was created by displacement of each dot by the appropriate amount at the monitor refresh rate of 75 Hz.

#### Recordings

Extracellular recordings from the left hemisphere were conducted using tungsten microelectrodes (impedance 0.5–2 mΩ, Microprobe and FHC). Electrodes were lowered through a recording chamber implanted on top of the parietal bone until reaching the approximate location of MT/MST. Single units were isolated with a window discriminator, and eccentricity, direction selectivity, and position of the electrode within the recorded area were determined. RFs of MT and MST neurons were mapped using a moving bar attached to the computer cursor. Upon implantation of the recording chamber and craniotomy to give access to area MT, we positioned an adaptor on top of the chamber containing a grid (Crist Instruments, TX). We then positioned a guide tube with an inner guide through the grid and penetrated the dura mater until about 2–3 mm from the point the guide tube touched the dura mater. We retract the inner guide and let the guide tube positioned on the grid. Then we used a Microdrive and positioned an electrode (from 70 to 200 Micrometers shank) inside the guide tube. We then advanced the electrode via a hydraulic Microdrive (Kopf Instruments, CA). We advanced the electrode slowly while stopping every 150 µm to listen to the neuronal activity. A first layer of gray matter was labeled by listening to activation of neurons, then a silent layer indicating the white matter and finally a second layer of activity. Once the second layer was identified we stopped advancing the electrode and labeled that layer as putative MT. We then advanced the electrode in steps of 50 µm until recording action potentials. Then, we started the mapping procedure.

Mapping was conducted using a virtual bar attached to the computer mouse cursor. Systematic mapping of the entire visual field in 8 different orientations, 16 directions was conducted per quadrant while listening to the pattern of neuronal activity and visualizing the action potentials through an oscilloscope while the animal fixated a central fixation point. When we detected an increase in the firing, we concentrated on that area of the screen and performed an estimation of the RF boundaries using the bar. We drew the boundaries on a transparency we attached to the computer screen. Once boundaries were estimated, we positioned RDPs inside the RF moving in the neuron’s putative preferred and anti-preferred directions. We increased the size of the RDP (diameter) in order to estimate where the RF surround started. Then, we measured direction and speed tuning of the neuron using an array of stimuli (8 different directions: 0, 45, 90, 135, 180, 225, 270, and 330°, 0° was the horizontal and to the right direction relative to the animal, speed: 2, 4, 8, 12, 16, and 32°/s). We then estimated the preferred direction and speed of the neuron through online display and fitting Gaussian tuning curves. We also used spiral stimuli to measure the responses of neurons. The spiral stimuli had similar average speeds as the linear motion stimuli and were as follows: expansion, clockwise rotation + expansion, clockwise rotation, clockwise rotation + contraction, contraction, contraction + counter-clockwise rotation, counter-clockwise rotation, and counter-clockwise rotation + expansion [67]. Neurons were classified as MT if the receptive fields were limited to the contralateral visual hemifield, and if they were better tuned to linear than to spiral motion and the receptive field size did not cover an entire hemifield or quadrant. We recorded only from those neurons showing clear direction selectivity during initial mapping. Throughout this process, we recorded from 107 MT/MST neurons (75 MT and 32 MST neurons).

#### Task

The animals were trained to selectively attend to a cued stimulus (the target), while directing gaze to a fixation point (Fig. 1A). One of the RDPs always moved in the neuron’s preferred direction (preferred pattern) and the other could move in one of 12 different directions from trial to trial (tuning pattern, in steps of 30°). There were three different experimental conditions depending on which stimulus on the display was attended (Fig. 1B). The animals were cued to attend to (1) the preferred pattern (attend-preferred condition), or (2) tuning pattern (attend-tuning condition). Then, 200 ms after the animal foveated the fixation point, a stationary RDP appeared as an attentional cue, indicating the location of the target. Once the animal pressed a lever, the cue moved in the target direction for 400 ms and then disappeared to re-appear together with the distractor after an interval of 270 ms. The direction of either the target or distractor changed (15–25° for 110 ms) within a time window of 270–1130 ms after target and distractor onset (see [12] for the detailed information regarding the change probability distribution). The task for the animals was to covertly attend to the target pattern and release a lever only in response to a direction change in the target within a 250–700-ms response window after the direction change. The animals had to ignore changes in the direction of the unattended pattern, which happened in 50% of the trials. Direction change occurred in every trial except in the fixation conditions. (3) In the fixation condition, the animals attended to the fixation point and released a lever once they detected the color change of the fixation point while ignoring both RDPs in the periphery. Only correctly completed trials were rewarded with a drop of juice and included in the data analysis. Trials where the animals failed to maintain fixation or responded outside the reaction time window were discarded.

#### Data analysis

Neurons were included in the analysis if the number of data points in which they were recorded from was more than 6. As a result, 78 MT/MST neurons were analyzed (53 MT and 25 MST neurons). We computed average firing rates during the interval from 200 to 1200 ms after the onset of the two patterns, as a function of the tuning pattern’s direction relative to the direction of the preferred pattern. Responses after a direction change in the receptive field were excluded from the analysis. They were analyzed separately in the context of another study [68]. The responses of each neuron were normalized to the response when both RDPs moved in the neuron’s preferred direction in the attend-preferred condition and then averaged across neurons. The responses of both MT and MST neurons were pooled since the direction selectivity and tuning curve profiles were very similar between the two areas. Repeated-measures ANOVA and paired samples *t*-tests on the average normalized neuronal response were conducted to examine how neuronal responses varied depending on experimental conditions.

We fitted the single Gaussian and sum of two Gaussians models to neuronal responses, using the MATLAB curve fitting toolbox (Mathwork Inc., USA). The sum of two Gaussians model we used is equivalent to the difference-of-Gaussians model as the first Gaussian has a positive gain and the second Gaussian has a negative gain. We used the Akaike Information Criterion (AIC [69]) which penalizes model complexity (lower AIC value indicates better fit) to assess the relative quality of each model fit.

### Human behavioral experiments

#### Participants

Fourteen naïve participants (5 men, 9 women), between the ages of 20 and 30 years completed the experiment. They had normal or corrected-to-normal visual acuity, and normal color vision. Written informed consent was obtained from all participants and they were paid for their participation ($30 CAD per participant). The research was approved by York University’s Human Participants Ethics Review Committee.

#### Apparatus and stimuli

Experiments were conducted in a dark room. Participants sat 57 cm from a CRT monitor (21″ View Sonic G225f, 1280 × 1024, 85 Hz), and their heads were stabilized on a head and chin rest (Headspot, UHCOtech, Houston, TX). Participants wore an infrared eye tracker (Eyelink II, SR Research, 500 Hz, Mississauga, ON, Canada) monitoring the left eye position. Random dot patterns (RDPs) were created through MATLAB (MathWorks, Natick, MA) and the Psychophysics Toolbox [70, 71]. Experimental control was maintained by Presentation (Neurobehavioral Systems, Berkeley, CA).

An annular RDP consisted of two superimposed motion surfaces (RDP size = 15 dva (degree in visual angle) in diameter, inner aperture size = 6 dva in diameter, dot size = 0.15 dva, 75 dots per surface). The directions of the motion surfaces changed every trial and the dots in each motion surface moved in the same direction (100% coherent). Directional difference between the two surfaces systematically varied by 10 ~ 50° (10° step). Motion speed was either 3°/s or 6°/s and both motion surfaces moved in the same speed. Dots in one motion surface were red (luminance: 24.67 cd/m^2^), and those on the other surface were green (24.64 cd/m^2^) to make participants easily segregate them, without affecting direction repulsion [57].

#### Task

We tested participants under two experimental conditions: divided and focused attention. In the divided attention condition, typical motion repulsion was measured. The RDPs were presented for 2 s once participants fixated a white cross centered on a screen for 200 ms. Participants had to maintain the fixation until the RDPs disappeared; otherwise, an error message was presented, and the trial was randomly interleaved in the remaining trials. Participants were asked to view both motion surfaces equally to estimate their directions. During motion presentation, a brief directional shift (100 ms) on either motion surface could randomly occur in 80% of trials, and then it went back to the original direction. The amount of shift was randomly selected from the range between 30 and 40° when motion speed was 3°/s, and between 20 and 30° when motion speed was 6°/s to equalize the perceptual strength of directional shifts across different motion speed conditions. A shift could occur from 650 to 1100 ms after RDP onset. Participants were asked to ignore this directional shift while they viewed the RDPs. After motion presentation, a color cue (either red or green) appeared for 250 ms to indicate which motion surface was the target in that trial. Each color cue was presented equally throughout the experiment. Participants reported the motion direction of the target surface by clicking along a white circular outline.

In the focused attention condition, after maintaining central fixation for 200 ms, a color cue appeared before the RDPs were presented to indicate which motion surface should be attended. Participants were required to attend only to the cued motion surface (target) while ignoring the other surface. To make sure whether they selectively attended to the target surface, they clicked the right mouse button within 1 s after the onset of a brief directional shift. The directional shift could occur only on the target surface. If there was no shift, participants did not respond and waited until they viewed the white circular outline. If participants missed the shift, responded too late, or made a false alarm, an error message was presented, and the trial was discarded. They reported the motion direction of the target surface only when the attention task was successfully performed.

The attentional conditions were blocked, and participants performed both conditions twice in a random order. At the beginning of each attentional condition, participants were given 10 practice trials whose data were not used, and then, they performed 100 trials as the main experiment (200 trials for each attentional condition, in total). There was a mandatory “break time” after every 25 trials. Participants could have extra “break time” if they wanted.

#### Data analysis

We first sorted participants’ direction judgment responses to reduce variability in data [57]. A correct direction judgment should fall within a range that extended from halfway between the two motion directions to 45° away from the motion direction of the target surface. Then, motion repulsion, defined by the difference between the reported (perceived) and the actual motion direction, was calculated. Since participants performed an additional attention task in the focused attention condition, only trials in which both directional shift detection and motion direction judgment were successful were included in the analysis. Motion repulsions in the two attentional conditions were compared to quantify the attentional modulation.

## Supplementary Information

**Additional file 1: Table S1.** Distribution of all neurons’ minimum responses. **Figure S1.** Histogram of Table S1. **Table S2.** Distribution of minimum responses of the neurons where the sum of two Gaussians model fits better. **Figure S2.** Histogram of Table S2. **Table S3.** Distribution of minimum responses of the neurons with the center-surround profile. **Figure S3.** Histogram of Table S3.

## Data Availability

All data generated or analyzed during this study are included in this published article, its supplementary information files, and publicly available repositories (https://doi.org/10.17605/OSF.IO/5QN74) [72].

## Abbreviations

MT: Middle temporal area
RDP: Random dot pattern
RF: Receptive field
MST: Medial superior temporal area
SEM: Standard error of the mean
ANOVA: Analysis of variance
*M*_diff_: Mean difference
SE: Standard error
BH: Benjamini–Hochberg procedure
AIC: Akaike Information Criteria
PV: Parvalbumin positive cells
DVA: Degree in visual angle
