# Supplementary material for: Attention to visual motion suppresses neuronal and behavioral sensitivity in nearby feature space
Source: BMC Biol. 2022 Oct 5;20:220. doi: 10.1186/s12915-022-01428-7 (PMC9535987; doi:10.1186/s12915-022-01428-7)
Supplement: Supplementary file 1 — Additional file 1: Table S1. Distribution of all neurons’ minimum responses. Figure S1. Histogram of Table S1. Table S2. Distribution of minimum responses of the neurons where the sum of two Gaussians model fits better. Figure S2. Histogram of Table S2. Table S3. Distribution of minimum responses of the neurons with the center-surround profile. Figure S3. Histogram of Table S3. [file 12915_2022_1428_MOESM1_ESM.docx]

**Additional file 1: Directional difference where individual neurons showed minimum responses**

First, we calculated the minimum responses of all neurons (n = 78), and plotted a graph of their locations (i.e., directional differences).

| Directional difference (absolute value) | Count* |
| --- | --- |
| 0 deg | 2 |
| 30 deg | 6 (3, 3) |
| 60 deg | 16 (10, 6) |
| 90 deg | 23 (9, 14) |
| 120 deg | 9 (4, 5) |
| 150 deg | 16 (11, 5) |
| 180 deg | 6 |

Table S1. Distribution of all neurons’ minimum responses. *Count = total number of minimum responses at each directional difference (when the directional difference has a negative value, and when it has a positive value)

Fig. S1 illustrates that minimum responses were mostly observed at around 60~90 deg (mean = 99.62 deg, SD = 45.48 deg).


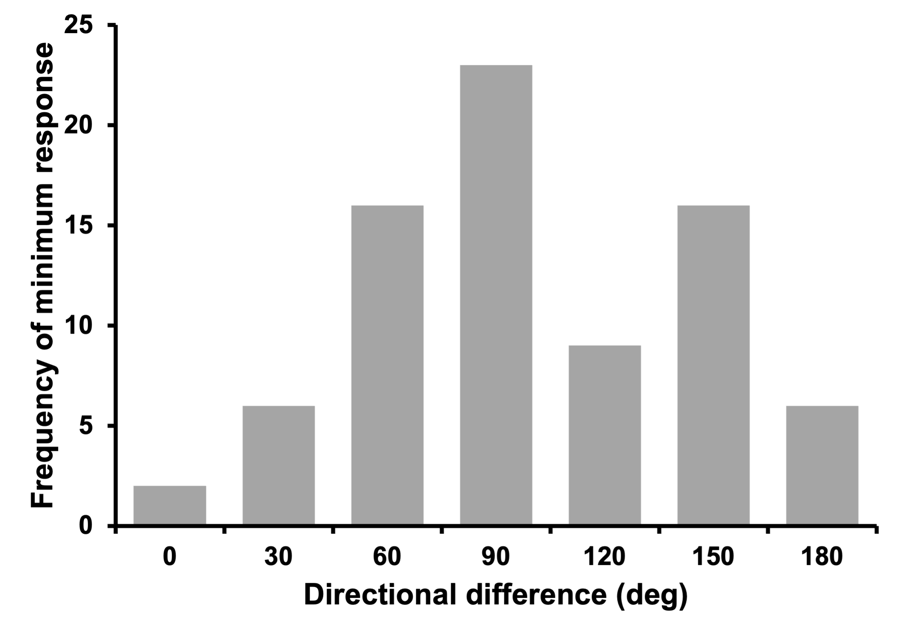


Fig. S1. Histogram of Table S1.

Second, we did the same analysis using the neurons where the sum of two Gaussians model fits better their responses than the single Gaussian model does (goodness-of-fit was measured by AIC, n = 38).

| Directional difference (absolute value) | Count |
| --- | --- |
| 0 deg | 1 |
| 30 deg | 3 (3, 0) |
| 60 deg | 13 (8, 5) |
| 90 deg | 10 (4, 6) |
| 120 deg | 5 (3, 2) |
| 150 deg | 4 (2, 2) |
| 180 deg | 2 |

Table S2. Distribution of minimum responses of the neurons where the sum of two Gaussians model fits better.

Again, minimum responses were mainly observed at 60~90 deg (mean = 87.63 deg, SD = 42.07 deg).


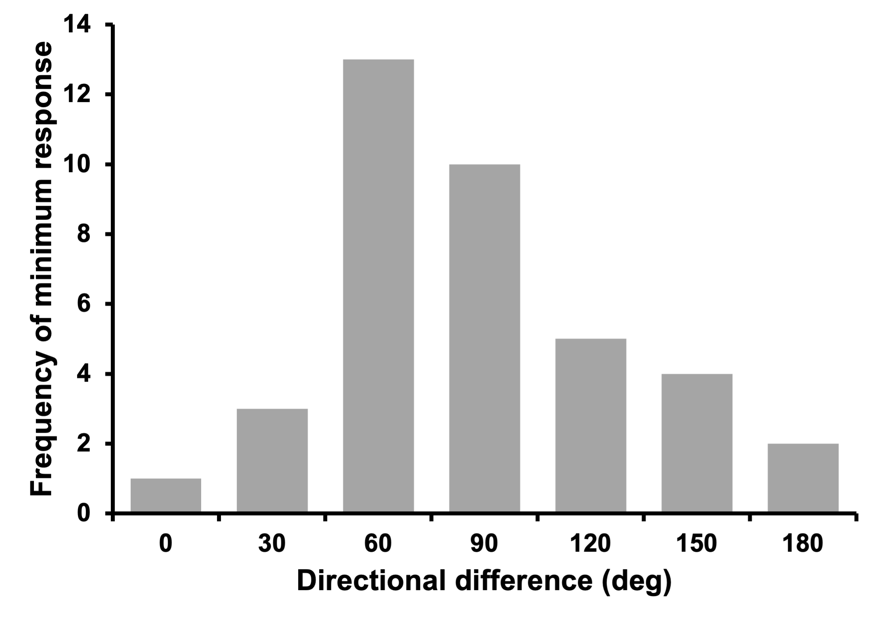


Fig S2. Histogram of Table S2.

Lastly, we selected the neurons based on their tuning curves’ shapes (n = 43). They showed one local maximum and two local minima when the sum of two Gaussians model was fit, representing the center-surround profile.

| Directional difference (absolute value) | Count |
| --- | --- |
| 0 deg | 0 |
| 30 deg | 1 (0, 1) |
| 60 deg | 15 (9, 6) |
| 90 deg | 18 (8, 10) |
| 120 deg | 5 (2, 3) |
| 150 deg | 2 (2, 0) |
| 180 deg | 2 |

Table S3. Distribution of minimum responses of the neurons with the center-surround profile.

It shows the similar result (mean = 88.60 deg, SD = 32.70 deg).


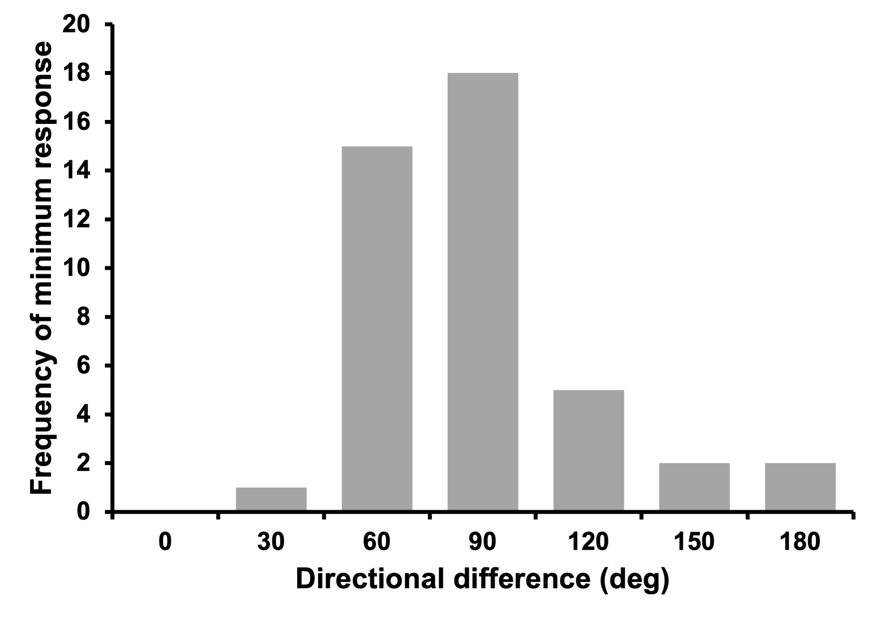


Fig S3. Histogram of Table S3.

Overall, these analyses support the existence of attentional surround suppression in the motion direction domain.
